# Average Duration of Human Life

**Published:** 1856-03

**Authors:** 


*The Average Duration of Human Life in Various Countries and at Different Epochs.*

In France a sixth of the population die at the end of the first year; a fifth at the end of 2 years; a third at the end of 14 years; the half at the end of 42 years; the three-fourths at the end of 69 years; the four-fifths at the end of 72 years, and the five-sixths at the end of 75 years.

Before 1789, Duvillard calculated that of 100 men, 50 live to 20 years; but since 1789 there is a marked improvement, and the observations of Bienaymè, from 1823 to 1831, prove that 60, and not 50, is the true proportion. Demontferrand says that of 100 men, 7 live to 80 years, 2 to 85, 1 to 89; and that in a million of our race there are only 640 nonagenarians (from 90 to 99 years.) Matthieu stated the number at 491, and of these only 9 reached the age of 97 years, and 4 that of 99.

Centenarians, according to Duvillard and Demontferrand, exist in the proportion of 2 in every 10,000. There are, however, privileged countries. Thus, at Carlisle, Milne found 9 in the 10,000. At most one dies in Paris every year.

Benoiston de Châteauneuf having examined 15 million lives, finds that 44 in 100 live to the age of 30; 23 to 60; 15 to 70; 4½ to 80, and 44-73ds to 90.

At present the average duration of life in France appears to be 39 years 8 months. Twenty years ago, Bienaymé valued it only at 36 years, and Demontferrand represented it at 33 years 8 months. In 1817 it was only 31 years 3 months; before 1789, according to Duvillard, 28 years 9 months; and Villermé has established that in Paris during the 18th century it was 32 years; in the 17th, 26 years, and only 17 years in the 14th century.

In France but one septagenarian is found among every 33 individuals; one octogenarian in every 160, and but one nonagenarian in 1900. Of these last there are nearly 17,500. Matthieu, however, computes that in every 174 persons there is one octogenarian, and one nonagenarian in every 1740.

At Geneva the average duration of life was 18 years 5 months in the 16th century: 23 years 4 months in the 17th, and from 32 ao 33 years in the 18th; from 1815 to 1826, it has risen to 38 years 10 months.

At present in France, as we have seen, the average duration of life is 39 years 8 months, that is to say, on our birth we have before us 39 years and 8 months of probable existence; at 4 years, a period when all the favorable chances are united, we have 49 years 4 months; according to Deparcieux, we have only 40 years and 3 months at 20 years of age; 34 years and 1 month at 30 years of age; 27 years 6 months at 40; 20 years 5 months at 50; 14 years and 3 months at 60; 8 years and 3 months at 70; 4 years and 8 months at 80; and 1 year and 9 months at 90.

In 1840 the average duration of life in England was 38 years; in France 36 years and a half; in Hanover, 35 years 4 months; in Schleswig-Holstein, 34 years 7 months; in Holland 34 years; in the Duchy of Baden, 32 years 9 months; at Naples, 31 years 7 months; in Prussia, 30 years 6 months; in Wurtemburg, 30 years, and 29 years in Saxony.

Demontferrand has divided into three classes the departments where life is the longest, and also those where it is briefest. In the first class, which is that of the longest livers, there are 28 departments: Calvados, Gers, Basses-Pyrénées, Cantal, Charente, Orne, Lot-et-Garonne, Lot, Maine-et-Loire, Aveyron, Gironde, Lozére, Deux-Sèvres, Manche, Tarn-et-Garonne, Doubs, Mayenne, Dordogne, Creuse, Loire-Inférieure, Eure, Vienne, Haute-Marne, Indre-et-Loire, Haute-Loire, Ariége, and Haute-Garonne. The average duration of life for example, is 44 years 7 months in the Calvados and the Lot-et-Garonne.

The second class contains 33 departments: Jura, Puy-de-Dôme, Vendée, Sarthe, Charente-Inférieure, Corse, Seine-et-Oise, Somme, Oise, Tarn, Seine-Inférieure, Corrèze, Eure-et-Loire, Côte-d'Or, Pas de-Calais, Ardennes, Aube, Marne, Drôme, Allier, Vosges, Ille-et-Vilaine, Isère, Yonne, Var, Meurthe, Meuse, Aude, Laudes, Herault, Aain.

The third class comprehends the other 25 departments. In Finisterè and Pyrénées-Orientales, the average duration of life is only 28 years 2 months and 28 years 1 month. Females have the advantage; thus in every 100 of each sex, at 10 years there are 58 females and 53 males alive; at 20 years, 58 females and 48 males; at 50 years, 33 females and 30 men; at 60 years, 23 females and 23 males; at 70 years, 15 females and 13 males; at 80 years, 5 females and 4 males, according to Benoiston (de Châteauneuf); and although 17 boys are born for every 16 girls, the proportion is soon re-establised; thus at the end of one year for every 1000 children of each sex there are 848 girls and 823 boys alive.

These observations, made with the greatest possible accuracy, are curious in the extreme, and from them the legitimate conclusion is drawn that the average duration of life in Europe, and especially in France, is increasing every year.—*Moniteur—quoted in the Gazette des Hôpitaux, July* 14*th*, 1855.

### *Population and Mortality of England.*

"Mr. Finlaison estimates the population of England for the close of the seventeenth century at about five millions and a quarter of *souls*. It is to be presumed that, in fixing this amount, he has not been hypercritical in the matter of psychological qualification, and that it may therefore be taken for granted that this number expresses the sum total of unfledged bodies which moved about upon two legs in the land of the Angles at that time. At the middle of the nineteenth century, appointed enumerators found that the five millions and a-quarter had become, in round numbers, eighteen millions; that, in fact, the inhabitants of England (not taking Scotland into the account) had nearly quadrupled themselves in 150 years, besides sending countless crowds to people vast lands in the west and in the south. In the year 1851, it was found that London contained within its own precincts nearly half as many inhabitants as all the island held in 1701. On one memorable day—the 9th of October 1851, there were collected in a single hall erected in one of its parks, a 56th part of the full complement of English people that were alive in 1701. The Registrar-General has endeavored, in his report of the result of the recent census to furnish a definite idea as to what millions mean where population is concerned. He states that if every person living in Great Britain had only a square yard to stand upon, there would be still seven square miles of the crowd when all were collected together; or if every individual were planted out over the surface of the island, as cabbages are planted out in the best arranged market-gardens, each would be 103 yards away from his neighbors. Let the entire crowd of these animated cabbages be gathered from all the corners of the land, and be required to file through Temple Bar in a column of four deep, and moving at a quick marching step, it would take three months of twelve hour days (Sundays being allowed for rest) to get the entire living stream through; if a single individual were to undertake to pass them through a gate, as turnpike men do flocks of sheep, enumerating them at the rate of one every second as they went, he would be a year and a-half, working twelve hours a day (exclusively of Sundays) before he finished his task. Now, it has been ascertained that this vast mass of people is at the present time occupied with doubling itself every fifty-two years and a-half; so that if the same state of affairs were to continue without interruption or change until the year 2534, the inhabitants of Great Britain, instead of each having the space of 180 square yards to himself, would all touch each other by the elbows. There would be just standing room for all, but no one must think of moving. Britannia, with its eleven degrees of longitude and ten degrees of latitude, would then look from the ocean like a huge rock covered by its impenetrable crowd of penguins or boobies. Such, indeed, under these circumstances, would be the prospects of our favored land.

These considerations are surprising in themselves; but there is an important fact connected with them, that greatly enhances the wonder with which they are contemplated when it is brought prominently into view. This rapid augmentation in the ranks of the population has gone on, not only in despite of a constant emigration to foreign lands (more than two millions and a-half of individuals emigrated between the years 1821 and 1851,) but also in the face of the yet more serious drain from the operation of the ordinary conditions of mortality. More than 1000 people died every day in England and Wales during the year 1850.

Of the eleven millions who were alive in 1801, eight millions had passed to the silent tomb, before those eleven millions had multiplied into twenty-one millions in 1841. Before the twenty-one millions can become forty-two millions, which they would do by 1904, under the present rate of increase, at least thirty millions will have been swept from the earth, by either premature or natural decay. By that time nineteen millions of British subjects will also have found a foreign home, even if the rate of migration does not increase with augmenting numbers. But emigrants double their ranks in considerable less time than half a century, on account of their consisting almost exclusively of individuals included within the reproductive periods of life. Hence, the nineteen millions of British emigrants scattered over the world will be really more than thirty-eight millions in their new houses. If then, the existing state of things be continued without modification for another half-century, it may fairly be assumed that the twenty-one millions at present inhabiting our island will have changed themselves into a hundred millions of souls. In reality, it is known that this rate of multiplication does not take place over any large area of the earth, on account of various modifying and repressing influences that are continually at work. Thus, although the population of Great Britain nearly doubled itself in the half century that preceded 1851, there was an absolute diminution of numbers in the year 1850, if taken alone. In that year the excess of births over deaths amounted to 224,000; but 280,000 emigrated during the same period. There were 56,000 more departures than arrivals; and the British field of human plants was therefore thinned out rather than thickened, in this interval. Still the abstract fact remains, that the real reproducing power of man in a state of civilization is expressed by a fivefold increase in half-century periods; at the lowest estimate, when every circumstance is favorable to the result, twenty millions made into a hundred millions, living and dead, may be taken to be a rude expression of the powers of human fertility.

The numbers in any population, where the births represent all arrivals, and the deaths all departures, must always be in a certain ascertainable ratio to the mean duration of individual life, taken in connection with the amount of the additions by birth in each year. The number of yearly births multiplied by the mean length of individual life in years, under such circumstances always gives the sum of the population. Thus, in 1851, when England and Wales contained eighteen millions of people, the births amounted to about 600,000; and accordingly the average duration of individual life would have been fifty years, if there had been no other drain upon the numbers than that produced by death (600,000 X 30 = 18 millions). In reality, however, as the eighteen millions represented a result lowered by the drain of emigration, as well as by that of death, the true value of individual life was considerably higher. It is only under circumstances of great exposure to deleterious influences that the expectation of life sinks so low as this in England. In the worst managed workshops in London, where men of intemperate habits sit long in a close, impure atmosphere, the average duration of life does not rise above thirty-two years; but in cities generally it reaches thirty-eight years, and in the open country, as high as fifty-eight years.—*Virg. Med. and Surg. Journal, from Mann's Philosophy of Reproduction.*

